# Different Patterns in Ranking of Risk Factors for the Onset Age of Acute Myocardial Infarction between Urban and Rural Areas in Eastern Taiwan

**DOI:** 10.3390/ijerph18115558

**Published:** 2021-05-22

**Authors:** Hsiu-Ju Huang, Chih-Wei Lee, Tse-Hsi Li, Tsung-Cheng Hsieh

**Affiliations:** 1 Institute of Medical Sciences, Tzu Chi University, Hualien 97004, Taiwan; 102353112@gms.tcu.edu.tw (H.-J.H.); lcw@mail.tcu.edu.tw (C.-W.L.); 2 Department of Physical Therapy, Tzu Chi University, Hualien 97004, Taiwan; 3 School of Medicine, College of Medicine, Taipei Medical University, Taipei 11041, Taiwan; b101105030@tmu.edu.tw; 4 Doctoral Degree Program in Translational Medicine, Tzu Chi University and Academia Sinica, Hualien 97004, Taiwan

**Keywords:** acute myocardial infarction, urban areas, rural areas, reversible risk factors, smoking, obesity, dyslipidemia, hypertension

## Abstract

This cross-sectional study aimed to investigate the difference in ranking of risk factors of onset age of acute myocardial infarction (AMI) between urban and rural areas in Eastern Taiwan. Data from 2013 initial onset of AMI patients living in the urban areas (n = 1060) and rural areas (n = 953) from January 2000 to December 2015, including onset age, and conventional risk factors including sex, smoking, diabetes, hypertension, dyslipidemia, and body mass index (BMI). The results of multiple linear regressions analysis showed smoking, obesity, and dyslipidemia were early-onset reversible risk factors of AMI in both areas. The ranking of impacts of them on the age from high to low was obesity (β = −6.7), smoking (β = −6.1), and dyslipidemia (β = −4.8) in the urban areas, while it was smoking (β = −8.5), obesity (β= −7.8), and dyslipidemia (β = −5.1) in the rural areas. Furthermore, the average onset ages for the patients who smoke, are obese, and have dyslipidemia simultaneously was significantly earlier than for patients with none of these comorbidities in both urban (13.6 years) and rural (14.9 years) areas. The findings of this study suggest that the different prevention strategies for AMI should be implemented in urban and rural areas.

## 1. Introduction

Cardiovascular disease (CVD) is a global health problem that has reached epidemic scale in developed and developing countries. Despite the decline in mortality caused by CVD, the burden of disease remains high [1]. The cost associated with CVD in Japan was $56.1 billion in 2013 [2]. In Taiwan, the average total medical costs of the first two years following the onset date of MI, angina, and stroke date were NT$12,671, NT$3324, NT$6863, respectively [3]. The annual total cost of CVD in the United States was estimated at $351.2 billion in 2014–2015 according to the AHA 2019 Heart Disease and Stroke Statistics [4]. The WHO recently indicated that the global burden of cardiovascular mortality is expected to predominantly occur among developing countries over the next decades. The large scale of studies for investigating key public health measures, such as geographic variations (e.g., urban and rural areas), traditional and potential newly discovered risk factors, and gaps for improvement in healthcare prevention are suggested [1]. A review of CVD burden in the United States between 1990 and 2016 revealed significant discrepancies among different states. The researchers concluded that further research into the differences between urban and rural areas was needed [5].

The Framingham Heart Study is the most influential epidemiological survey of CVD in the United States [6]. In the study, the researchers tracked the epidemiology of large communities in Framingham, Massachusetts. They found that hypertension, diabetes, dyslipidemia, smoking, and obesity [6,7] were the independent potential risk factors of CVD. The ranking of risk factors in different countries varies considerably [8]. The top three risk factors in the Framingham Heart Study were hypercholesterolemia, hypertension, and smoking [6,7]. Kawano et al. (2006) examined 1353 patients with acute myocardial infarction (AMI) and found that the top three risk factors for men were sequentially hypertension, smoking, and diabetes, and those for women were sequentially smoking, diabetes, and hypertension [9]. The Chin-Shan Community Cardiovascular Cohort (CCCC) was the first community cohort study on CVD in Taiwan. The researchers tracked 3602 respondents over 35 years old residing in the Chin-Shan Community for five consecutive years starting in 1990. They found that hypercholesterolemia, smoking, and hypertension were the key factors for men, and hypercholesterolemia and hypertension were the key factors for women [10]. In addition to the differences in the ranking of traditional risk factors associated with coronary heart disease in different countries, the findings of a study on the incidence rate of myocardial infarction between coastal and inland residents residing in the Yamagata Prefecture of Japan indicated that in coastal areas, young men had an increased AMI incidence, while older females exhibited an increased AMI incidence in urban inland areas. Moreover, the increase in AMI incidence in males in the coastal area was associated with an increasing prevalence of dyslipidemia [11]. A Taiwanese study of the risk factors associated with the incidence of seasonal AMI in the tropical and subtropical climate zones in 6897 AMI patients between 1997 and 2011 showed that older adults 65 years of age and older residing in subtropical regions and patients diagnosed with diabetes mellitus (DM) or dyslipidemia were at higher risk of developing seasonal AMI. However, similar results were not exhibited in the patients residing in tropical regions [12]. This study highlighted that the risk factors of myocardial infarction differed in the different regions of a country. Subsequently, different regions may have different effects on the age of AMI onset.

Increasing age is a recognized risk factor of AMI [13]. Yao et al. [14] indicated that age is also a factor for a poor 6-month prognosis in AMI patients. Yao’s study showed that patients with a poor prognosis were about 10 years older. The poor prognosis of AMI certainly will increase the disease burden in treatment and caring. The characteristics and incidence rate of coronary heart disease in young people are not as prevalent as found in older adults. However, coronary heart disease can cause devastating consequences for young patients and their families [15]. The findings of recent studies show significant differences in the risk factors, clinical characteristics, coronary angiography features, and prognostic outcomes of young and old AMI patients. A meta-analysis [16] on the differences between young and old AMI patients showed that smoking, family history of coronary heart disease, obesity, and drinking are the main risk factors for young AMI patients. Results also showed that young AMI patients were less likely to have a history of hypertension and diabetes. Other studies have found that young AMI patients were more likely to die prematurely or develop long-term disabilities, while older AMI patients had worse prognoses.

Eastern Taiwan occupies 22.7% of Taiwan’s land area with the Pacific Ocean lying to the East and the Central Mountain Range lying to the West. Eastern Taiwan is cut longitudinally by the Hai’an Range, dividing the area into flat urban regions and mountainous rural regions. Culture, work patterns, living habits, distribution of medical resources, and unhealthy behaviors vary in the different regions [17,18]. Few studies have examined the differences in the factors influencing the onset age of AMI in urban and rural regions. In this study, we examined the characteristics of AMI patients admitted between 2000 and 2015 to identify and rank the factors influencing the onset age of myocardial infarction in urban and rural areas and compare the similarities and differences of the factors in urban and rural areas. The findings of this study can serve as a reference for public health departments to develop adaptable preventative and treatment policies for AMI.

## 2. Materials and Methods

### 2.1. Study Cases

A cross-sectional was conducted at Hualien Tzu Chi hospital which is the largest scale hospital in eastern Taiwan. Most AMI patients in eastern Taiwan were transferred to this hospital. A total of 2013 initially diagnosed AMI patients from January 2000 to December 2015 were recruited in the current study and divided into two groups according to their living area: urban group (n = 1060) and rural group (n = 953). AMI was diagnosed by cardiovascular specialists based on characteristic clinical history, serial changes on the electrocardiogram, and an increase in cardiac enzymes. This study was approved by the Research Ethics Committee at the Buddhist Tzu Chi General Hospital (IRB106-118-B).

### 2.2. Risk Factors

The conventional risk factors for AMI including sex, smoking, diabetes, hypertension, dyslipidemia, and obesity were collected from medical records. The following criteria were used to define the physiological risk factors: (1) Diabetes: Fasting blood sugar ≥126 mg/dL by US CDC guideline [19] or a history of previous treatment (2) Hypertension: Systolic blood pressure ≥140/90 mmHg by US CDC guideline [20] or a history of previous treatment. (3) Dyslipidemia: The International Classification of Disease, Ninth Revision, Clinical Modification (ICD-9-CM) diagnosis codes of 272.x. [21] (4) Obesity: BMI was calculated as weight (kg)/square of height (m^2^). The Taiwan BMI classification [22] was used to define the weight status of the study subjects: obesity (BMI ≥ 27 kg/m^2^).

### 2.3. Region

The definitions of urban and rural areas are as follows: (1) Urban areas: Hualien City, Ji’an Township, Taitung City, Beinan Township; (2) Rural areas: Xiulin Township, Wanrong Township, Zhuoxi Township, Haiduan Township, Yanping Township, Daren Township, Jinfeng Township, Xincheng Township, Shoufeng Township, Fenglin Township, Guangfu Township, Ruisui Township, Yuli Township, Fuli Township, Chishang Township, Guanshan Township, Luye Township, Fengbin Township, Changbin Township, Chenggong Township, Donghe Township, Taimali, Dawu Township, Lvdao Township, and Lanyu Township.

### 2.4. Statistical Analysis

The data are expressed as the mean with standard deviation and the frequency with the ratio for continuous variables and categorical variables, respectively. Students’ tests for continuous variables and chi-square tests for categorical variables were used to evaluate the differences between the two groups. If the distribution of continuous variables was skewed evaluated by the Kolmogorov-Smirnov test, the Mann-Whitney test was used. In this case, the median with interquartile range (IQR) was provided. In order to investigate the difference of risk factors for the onset age of AMI between urban and rural areas, three multiple linear regression model analyses with the dependent variable of the onset age of AMI were performed. In the first regression model, area (urban vs. rural), conventional risk factors, and the interaction term between area and each conventional risk factor were included as the predictors. The significance of the interaction term indicated significant different effects of the corresponding conventional risk factor on the onset age of AMI. In order to evaluate the predictors for each of the areas, in the other two regression models (2nd and 3rd models), only the conventional risk factors were included as the predictors, one for urban’s patients and the other for rural patients. All *p* values were two-sided with a value < 0.05 considered statistically significant. Statistical analysis was performed with SPSS for Windows version 21.0 (IBM, Armonk, NY, USA)

## 3. Results

Table 1 summarizes the onset age and distribution of conventional risk factors of the urban and rural AMI patients during 2000–2015 in Hualien. Among the total of 2013 patients with AMI in eastern Taiwan, the average onset age of AMI was 66.9 years, 67.5% of which were male. The three highest prevalence of the conventional risk factors were hypertension (69.7%), diabetes (42.5%), and smoking (38.7%). The average onset age of AMI in the urban areas was 1.4 years earlier than the rural areas (66.3 ± 14.0 vs. 67.7 ± 13.4, *p* = 0.019). The proportion of males with AMI in the urban areas was significantly higher than the rural areas (71.5% vs. 63.0%, *p* = 0.001). Among the reversibly conventional risk factors, the prevalence of hypertension (70.2% vs. 69.3%) and diabetes (41.1% vs. 44.1%) were the two highest ones in both the urban and rural areas without significant difference between the areas. Both the prevalence of smoking (40.0% vs. 37.4%) and obesity (30.3% vs. 29.2%) did not differ significantly between the urban and rural areas, while the prevalence of dyslipidemia (33.7% vs. 38.1%, *p* = 0.039) in urban areas was significantly lower than rural areas.

**Figure 1:**
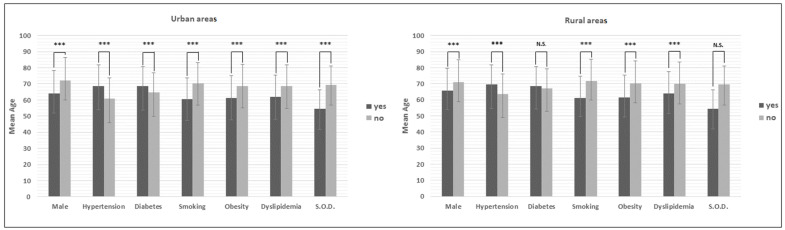
The onset age of AMI with and without conventional risk factors by area. N.S.: no significant difference (*p* ≥ 0.05). ***: *p* < 0.001. Error bar: standard deviation.

Table 2 and Figure 1 presented the mean onset age of AMI with and without conventional risk factors for the urban and rural areas, respectively. In the urban areas, the mean onset age of AMI for the patients with hypertension (68.7 years) or diabetes (68.5 years) was 8.0 or 3.8 years significantly later than the patients without hypertension (60.7 years) or diabetes (64.7 years). In comparison, the mean onset ages of male patients (64.0 years), the patients with dyslipidemia (62.0 years), smoking (60.5 years), or obesity (61.3 years) were 8.2, 6.5, 9.7, or 7.2 years significantly earlier onset age of AMI than the female patients (72.2 years), the patients without dyslipidemia (68.5 years), smoking (70.2 years), or obesity (68.5 years).

Similar results were observed in the rural areas except there was no significant difference in the mean onset ages of AMI between the patients with and without diabetes (68.5 vs. 67.1 years). The difference of mean onset ages of AMI reached significance for the male vs. female patients (65.8 vs. 71.0 years), the patients with or without hypertension (69.5 vs. 63.7 years), the patients with or without dyslipidemia (64.0 vs. 70 years), the patients with or without smoking (61.2 vs. 71.6 years), and the patients with or without obesity (61.5 vs. 70.3 years), respectively. Table 2 further compared the onset ages of AMI between the patients with the three reversibly risk factors (smoking, obesity, and dyslipidemia) simultaneously and the patients without any of them, the results showed that the onset age of patients with smoking, obesity, and dyslipidemia simultaneously was more than 13 years earlier than the patients without any of them in both urban (54.4 vs. 68.0) rural areas (54.5 vs. 69.4). It indicated the additive effect of these risk factors on the onset age of AMI.

Comparing the mean onset ages of AMI in Table 2 with the overall onset ages shown in Table 1, the onset age of AMI for the patients with smoking, obesity, or dyslipidemia were also earlier than the overall onset ages in both urban and rural areas. The results indicated that in both urban and rural areas, dyslipidemia, smoking, and obesity were the early onset AMI reversible conventional risk factors, while hypertension and diabetes were the late-onset reversible conventional risk factors of AMI.

Table 3 summarized the results of multiple linear regression analysis for comparing the predictors for the onset age of AMI between urban and rural areas. The onset age of AMI for male patients was significantly earlier than female patients in both the urban (β = −4.5) and rural (β = −2.2) areas. The onset age of AMI for the patients with hypertension was significantly later than those without hypertension in both the urban (β = 5.7) and rural (β = 4.5) areas. The significant reversible conventional risk factors in the urban areas were obesity (β = −6.7), smoking (β = −6.1), dyslipidemia (β = −4.8) ranked from high to low by the corresponding regression coefficient. For the rural areas, there were the same reversible significant risk factors for the onset age of AMI but with different ranks ranked as smoking (β = −8.5), obesity (β = −7.8), dyslipidemia (β = −5.1). Obesity was the most influential risk factor for the onset age of AMI in the urban areas, while smoking was the most influential ones in the rural areas. The significant interaction effect between area and smoking indicated that the influence of smoking on the onset age of AMI in the rural areas (β = −8.5) was significantly different and higher than the urban areas (β = −6.1).

## 4. Discussion

This study is the first clinical survey in Taiwan to examine the risk factors associated with the onset age of AMI based on urban and rural settings. We examined AMI patients admitted to the only medical center in Eastern Taiwan between 2000 and 2015 to identify the risk factors associated with AMI and rank the factors influencing the onset age of myocardial infarction in urban and rural areas. The main findings are as follows: (1) The proportion of females with AMI in the rural areas was significantly higher than in the urban areas; (2) The risk factor trends between the urban and rural areas were similar. Hypertension and diabetes were late-onset factors, while smoking, obesity, and dyslipidemia were early-onset factors; (3) The ranking of early-onset risk factors in urban areas from high to low as obesity, smoking, and dyslipidemia, and that in rural areas as smoking, obesity, and dyslipidemia; (4) Smoking was the primary early-onset risk factor in Eastern Taiwan, particularly in the rural regions; (5) The onset age of AMI in patients with obesity was younger than that of patients without obesity regardless of region. Obesity was also the primary factor in urban areas; (6) Regardless of region, the onset age of AMI of patients with smoking, obesity, and dyslipidemia was at least 13 years younger than that of patients without the three factors.

Among CVDs, ischemic heart disease is the leading global cause of death [23]. A survey on CVD burden conducted in the United States between 1990 and 2016 found varying results in different states. The researchers recommended that future studies focus on urban and rural factors [5]. The findings in the current study showed that the average onset age of myocardial infarction among patients in Eastern Taiwan in the 15-year period was 66.9 years. Male patients accounted for 67.5% of all the patients examined. These results are similar to those recorded in other Asian countries such as South Korea [24] and Japan [25]. Taiwan also exhibits similar trends to the United States [5,26] and Europe [6,7] regarding the average age and sex of myocardial infarction. Comparing the sex distributions between urban and rural areas in the study, the proportion of females with AMI in the rural areas (37%) was significantly higher than the urban areas (28.5%). A retrospective study for the comparison of hospital Admission, treatments, and outcomes for ST-Segment–Elevation Myocardial Infarction (STEMI) between urban and rural areas in China [27] showed that the proportion of female patients with STEMI in rural areas were equal to or higher than urban areas in 2001 (29% vs. 29%), 2006 (28% vs. 29%), and 2011 (28% vs. 33%), and the significant increasing trend was observed. The results that the proportion of female patients with STEMI in rural areas were higher than in urban areas was similar to our study for female patients with AMI. These similar results indicated that a higher proportion of female patients with AMI might be associated with the region since the study populations in both studies were Chinese population.

A recent study on patients admitted to a hospital in Florence, Italy for the initial onset of myocardial infarction showed that hypertension was a key factor. In particular, the average age of onset for patients with hypertension was 70.1 years, while that for patients without hypertension was 7.1 years older [28]. A survey on 12,625 patients admitted for acute myocardial infarction in South Korea between 2011 and 2015 highlighted that the average age of onset for patients with diabetes was 65.1 years, while that for those without diabetes was 63.3 years [29]. In Taiwan, a previous study that examined the data of 1073 coronary heart disease patients recorded between 1997 and 2003 in the Eastern Taiwan Integrated Health Care Delivery System of Coronary Heart Disease (ET-CHD) [30] reported that female myocardial infarction patients with diabetes (66.5 years) had an average onset age of 1.2 years less than those without diabetes (67.7 years). For men, the difference was 2.0 years. In the current study, we performed a regional analysis of patients in Eastern Taiwan between 2000 and 2015 and found a declining trend in the average onset age of myocardial infarction among patients without diabetes in urban and rural areas. These results are consistent with those found by studies in other countries [28,29,31]. The findings of this study also highlighted that the average onset age of myocardial infarction in AMI patients with hypertension or diabetes was later than that in AMI patients without hypertension or diabetes, regardless of region. Therefore, hypertension and diabetes can be confirmed as the late-onset risk factors of myocardial infarction.

A 30-year-long correlation analysis of dyslipidemia and mortality rate in 13,680 acute myocardial infarction or acute compensatory heart failure patients in the United States found that the average onset age of myocardial infarction in patients without dyslipidemia (71.3 years) was 4.3 years later than that in patients with dyslipidemia [32]. The findings of a study on the relationship between dyslipidemia and the incidence of patients with myocardial infarction residing in the coastal and inland areas of Yamagata Prefecture of Japan showed that the prevalence of dyslipidemia in AMI patients residing in coastal areas was lower than that in patients residing in urban and rural areas [11]. In this study, we found that the prevalence of dyslipidemia in AMI patients was 35% higher in urban areas than in rural areas and that dyslipidemia was the third most influential factor to the average onset age of AMI in both urban and rural areas. The average onset age of AMI in patients with dyslipidemia is lower than that of patients without dyslipidemia in both urban and rural areas. Therefore, early dyslipidemia prevention and treatment and detailed case management are recommended.

A report on 10,142 patients from 15 hospitals recorded in the Japan Cardiovascular Database (JCD) between 2008 and 2013 indicated that the obesity rate was 6.3% (n = 635) and that most of the patients with obesity were young. The average onset age of myocardial infarction in patients with obesity was 59.2 years, which was 10.2 years younger than that in patients without obesity (69.4 years) [33]. A survey of the initial onset of myocardial infarction in 28,742 patients in Malaysia produced similar results [34]. A study conducted in France yielded similar results [35]. The trends in Eastern Taiwan are similar to those of other studies. AMI patients with obesity exhibited an earlier onset of AMI than those without obesity. In this study, the average age of onset of AMI in patients with obesity was lower than that in patients without obesity (>6.7 years) in both urban and rural areas. Moreover, obesity is the leading factor influencing the average onset age of AMI in urban areas.

Smoking is a typical risk factor in western and Asian countries. A study on 324 AMI patients admitted to a regional heart institute in the United Kingdom reported that the average age of onset AMI for patients who smoked (55 years) was ten years less than that for patients who did not smoke (65 years) [36]. Similar results were obtained in a study conducted by researchers at the School of Medicine, Kumamoto University, Japan, which showed the age of onset AMI for smokers (59 years) was 16 years less than that for non-smokers (75 years). A study of the risk factors of male AMI patients in Eastern Taiwan between 1992 and 2006 showed that the number of smokers in the AMI group was higher than that in the normal coronary artery group, suggesting that in Eastern Taiwan, smoking is a key factor of AMI [37]. In this study, we found that smoking was the second most important factor affecting the early onset of AMI in urban areas. In rural areas, smoking was the most important factor affecting the early onset of AMI. The average age onset of AMI for smokers in the urban and rural areas was 6.1 years and 8.5 years earlier than that for non-smokers, respectively. An analysis of the interaction between smoking and region revealed that the effects of smoking on average age of onset was significantly greater in rural areas than urban areas.

### From the Perspective of Eastern Taiwan

The results of the current study indicated that obesity was the first and second most influential factor for the earlier onset age of myocardial infarction in urban areas and rural areas of Eastern Taiwan, respectively. Although the government is actively promoting Taiwan’s Obesity Prevention and Management Strategy, the statistics published in the 2013 to 2016 Nutrition and Health Survey in Taiwan (NAHSIT) highlighted that the prevalence of overweight and obese adults reached 45.4%. In terms of Taiwan, compared to a prevalence rate of 32.7% 20 years ago and 43.4% eight years ago, and rate of obesity has continued to increase steadily. The reasons for obesity in Taiwan include insufficient physical activity, sedentary lifestyle, and preference for high-sugar or high-calorie diets. In urban areas, the reasons might include inaccessibility of fresh food, large families, financial hardship, and excessive intake of alcohol and processed foods [38]. An analysis of data between 2000 and 2015 in our study showed that obesity is a longstanding risk factor for earlier onset age of AMI in Eastern Taiwan. The age of onset of myocardial infarction in obese patients is decreasing, particularly in urban areas. The government must review and revise relevant policies to ensure the enforcement of effective prevention strategies in urban areas.

At least 27,000 annual deaths in Taiwan are associated with smoking. Smoking is extremely detrimental to individuals, their families, and society as a whole. Since the ratification of the revised Tobacco Hazards Prevention Act in 2009, the number of smokers in Taiwan has dropped by 1.26 million. The smoking rate among adults dropped from 21.9% in 2008 to 14.5% in 2017, and that of young adults has shifted from an increasing trend to a decreasing trend. For example, the smoking rate among junior high school students dropped from 7.8% before ratification to 2.7% in 2017, and that of senior high school students dropped from 14.8% to 8.3% in 2017 [39]. The smoking rate is gradually declining. However, the findings of this study showed that smoking significantly affected the age of onset of AMI, particularly in rural areas, where the average age of onset of AMI in smokers is 8.5 years less than that in non-smokers. Therefore, smoking tobacco is hazardous and prevention of tobacco use remains a key agenda of the government, particularly in rural areas.

Dyslipidemia is the third most influential factor of the average age of onset of AMI in both urban and rural areas. Between 2000 and 2015, the average lifespan of Taiwanese people increased from 76.45 years to 80.19 years. In Eastern Taiwan, the average lifespan increased from 71.41 years to 75.94 years. The 16-year average lifespan was 73.76 years [40]. The findings of this study showed that between 2000 and 2015, the average age of onset for patients without all three factors was 72.8 years in urban areas and 75.3 years in rural areas. These results show that for patients without all three factors, the average age of onset and the average lifespan was relatively similar. In addition to controlling smoking and obesity, improving dyslipidemia is equally important.

According to findings of the ranking of risk factors for the onset age of AMI, obesity was the foremost early-onset risk factor in urban areas, followed by smoking and dyslipidemia. By comparison, smoking was the foremost early-onset risk factor in rural areas, followed by obesity and dyslipidemia. These findings highlight that different areas require different prevailing prevention issues and require different resources. In rural areas (dyslipidemia:smoking:obesity: β = −5.1:−8.5:−7.8), the effects of the various early-onset factors were far greater than those in urban areas (dyslipidemia:smoking:obesity: β = −4.8:−6.1:−6.7). Therefore, more prevention resources must be allocated to rural areas. Since the ratification of the revised Tobacco Hazards Prevention Act in 2009, smoking rates in Taiwan have gradually declined [16]. However, overweight and obesity rates have increased between 2013 and 2016 [41]. The prevalence of dyslipidemia in people with myocardial infarction has increased between 1999 and 2015 [42]. An extension of this study will be to examine the impact of the changing prevalence rates of the different risk factors on ranking. This study lacked data on patient’s exercise conditions at home. Therefore, we were unable to explain the effects of rising body mass indexes (BMI). In addition to BMI, the association between waist circumference/insulin resistance and age of onset will be our focus of subsequent research and items to be corrected in medical records.

## 5. Conclusions

The results showed that obesity, smoking, and dyslipidemia were the early-onset risk factors in urban areas, and smoking, obesity, and dyslipidemia were the early-onset risk factors in rural areas. These results highlight that different areas require different prevention initiatives The results of this study serve as a reference for advancing our understanding of the risk factors associated with AMI and formulating relevant countermeasures.

## Figures and Tables

**Table 1:** Distribution of conventional risk factors for urban and rural areas.

	All (n = 2013)	Urban (n = 1060)	Rural (n = 953)	*p*-Value ^#^
Age, mean ± SD	66.9 ± 13.8	66.3 ± 14.0	67.7 ± 13.4	0.019 *
Male, n (%)	1358 (67.5)	758 (71.5)	600 (63.0)	0.001 *
Hypertension, n (%)	1404 (69.7)	744 (70.2)	660 (69.3)	0.649
Diabetes, n (%)	856 (42.5)	436 (41.1)	420 (44.1)	0.183
Dyslipidemia, n (%)	720 (35.8)	357 (33.7)	363 (38.1)	0.039 *
Smoking, n (%)	780 (38.7)	424 (40.0)	356 (37.4)	0.224
Obesity, n (%)	599 (29.8)	321 (30.3)	278 (29.2)	0.586

SD: standard deviation; ^#^: *p*-value for comparing the urban and rural areas. *: *p* < 0.05.

**Table 2:** The onset age of AMI with and without conventional risk factors by area.

Variable.	Urban Areas (n = 1060)			Rural Areas (n = 953)		
	Yes Mean ± SD	No Mean ± SD	*p*-Value ^#^	Yes Mean ± SD	No Mean ± SD	*p*-Value ^#^
Male	64.0 ± 14.1	72.2 ± 12.1	<0.001	65.8 ± 13.8	71.0 ± 12.1	<0.001
Hypertension	68.7 ± 13.1	60.7 ± 14.7	<0.001	69.5 ± 12.3	63.7 ± 14.9	<0.001
Diabetes	68.5 ± 12.2	64.7 ± 15.0	<0.001	68.5 ± 12.0	67.1 ± 14.4	0.117
Dyslipdemia	62.0 ± 13.4	68.5 ± 13.9	<0.001	64.0 ± 13.5	70.0 ± 12.8	<0.001
Smoking	60.5 ± 13.1	70.2 ± 13.3	<0.001	61.2 ± 13.6	71.6 ± 11.7	<0.001
Obesity	61.3 ± 13.7	68.5 ± 13.6	<0.001	61.5 ± 13.9	70.3 ± 12.3	<0.001
With smoking, obesity, and dyslipidemia simultaneously	54.4 ± 11.1	68.0 ± 13.6	<0.001	54.5 ± 11.6	69.4 ± 12.7	0.332

SD: standard deviation; ^#^: *p*-value for comparing the urban and rural areas.

**Table 3:** Multiple linear regression analysis for association of conventional risk factors and the onset age of AMI for urban and rural areas.

Variables	Urban (n = 1060)			Rural (n = 953)			
	β	t	*p*-Value ^#^	β	t	*p*-Value ^#^	*p*-Value ^&^
Male	−4.5	−4.9	0.001 *	−2.2	−2.6	0.009 *	0.056
Hypertension	5.7	6.6	0.001 *	4.5	5.4	0.001 *	0.307
Diabetes	1.0	1.3	0.206	0.3	0.3	0.739	0.256
Dyslipidemia	−4.8	−6.0	0.001 *	−5.1	−6.5	0.001 *	0.826
Smoking	−6.1	−7.2	0.001 *	−8.5	−10.3	0.001 *	0.039 *
Obesity	−6.7	−8.2	0.001 *	−7.8	−9.5	0.001 *	0.350

β: unstandardized regression coefficient; t: t-value calculated based on *t*-test statistics for testing if β is statistically significantly different from zero; ^#^: *p*-value for testing β based on the regression model including the conventional risk factors as the predicators; ^&^: *p*-value for testing interaction effect between area and each conventional risk factor based on the model including area, the conventional risk factors, and the interaction term between area and each conventional risk factor; *: *p*-value < 0.05.

## Data Availability

The data that support the findings of this study are available on request from the corresponding author, T.-C.H. The data are not publicly available due to their containing information that could compromise the privacy of research participants.
